# The Mysuru stUdies of Determinants of Health in Rural Adults (MUDHRA), India

**DOI:** 10.4178/epih.e2018027

**Published:** 2018-06-23

**Authors:** Padukudru Anand Mahesh, Komarla Sundararaja Lokesh, Purnima Madhivanan, Sindaghatta Krishnarao Chaya, Biligere Siddaiah Jayaraj, Koustav Ganguly, Murali Krishna

**Affiliations:** 1 Department of Pulmonary Medicine, JSS Medical College and Hospital, JSS Academy of Higher Education and Research, Mysuru, India; 2 Department of Epidemiology, Florida International University, Miami, FL, USA; 3 Public Health Research Institute of India, Mysuru, India; 4 Institute of Environmental Medicine, Karolinska Institutet, Stockholm, Sweden; 5 Foundation for Research and Advocacy in Mental Health, Mysuru, India; 6 Faculty of Health and Social Care, Edgehill University, Lancashire, United Kingdom

**Keywords:** Lung diseases, Chronic obstructive pulmonary disease, Asthma, Spirometry, Biomass, Smoking

## Abstract

Between 2006 and 2010, in 16 randomly selected villages in rural areas of Mysore district, in south India, 8,457 subjects aged 30 and above were screened for symptoms of chronic respiratory disease. Of the 8,457 subjects, 1,692 were randomly invited for further evaluation of lung function and chronic obstructive pulmonary disease (COPD) by spirometry, and 1,085 of these subjects underwent lung function assessments for prevalent COPD and its risk factors. These 1,085 subjects, who were then aged between 35 and 80 years, constituted the Mysuru stUdies of Determinants of Health in Rural Adults (MUDHRA) cohort. Among other findings, threshold of biomass fuel smoke exposure suitable for use as a dichotomous risk factor for the diagnosis of chronic bronchitis was established, with a minimum biomass smoke exposure index of 60 found to be significantly associated with an elevated risk of developing chronic bronchitis. Five years later (between 2014 and 2016), 869 of the 1,085 participants were followed up with repeat lung function assessments for incident COPD and all-cause mortality. A subset of these participants (n=200) underwent blood tests for vitamin D levels, antioxidant activity, an assessment for anxiety and depression, and another subset (n=98) underwent a bioplex assay for 40 serum cytokines.

## INTRODUCTION

Chronic obstructive pulmonary disease (COPD) is a major cause of the global burden of morbidity and mortality and is projected to be the third leading cause of death by 2030 [1-3]. Of the more than 170 million COPD patients worldwide, more than 3 million people died in 2015. COPD accounts for 2.6% of global disability-adjusted life years. The burden of COPD is particularly high in rural areas of low- and middle-income countries, and it is the second leading cause of death in India [1,4,5] due to relatively high levels of smoking, ambient air pollution, exposure to biomass smoke, ozone, occupational particulate matter, and environmental tobacco smoke [4,6]. More than 3 billion people in the world are exposed to biomass smoke, compared to 1 billion smokers. We have shown previously that more than 90% of rural households used biomass fuels and that more than 50% of rural men smoked. Despite this, limited data exist on the prevalence of COPD and its burden in rural India [5].

In India, more than 70% of the population lives in rural areas [7]. The prevalence rates of COPD reported in population-based studies from rural India were not derived from standardised lung function assessments, but based on questionnaires [5,8-23]. Furthermore, in many studies, the participants were not randomly selected from the general population, which limits the generalizability of the findings. In those studies, the association of the dose of biomass smoke exposure with chronic respiratory disorders, particularly among non-smokers, was not examined. Those studies were primarily cross-sectional in design and were therefore limited to reporting prevalent COPD, and participants were not followed up to estimate the incidence of chronic respiratory disorders [5,8-23]. Therefore, the Mysuru stUdies of Determinants of Health of Rural Adults (MUDHRA) cohort was established to address some of these limitations, and specifically to examine the risk factors for prevalent and incident COPD among rural men and women in southern India.

COPD is characterized by local and systemic inflammation involving various chemokines and cytokines, and the pulmonary inflammation extends into the systemic circulation [24-28]. In Indian COPD patients, pulmonary and systemic inflammation is due to exposure to important risk factors, such as tobacco smoking (usually in men) or biomass smoke exposure (usually in women). Exposure to these risk factors leads to increased levels of pro-inflammatory cytokines and reduced levels of anti-inflammatory and homeostatic cytokines. The interaction of various cytokines and chemokines is insufficiently understood, especially in biomass smoke-related COPD, and preliminary data have shown that biomass smoke-related COPD is different from tobacco smoke-related COPD [29]. Some cytokines are associated with increased inflammation in COPD (interleukin [IL]-6 and tumour necrosis factor alpha [TNF-α]), the progression of disease (IL-2), or neutrophil recruitment (granulocyte-macrophage colony-stimulating factor, IL-8), whereas anti-inflammatory cytokines, such as IL-10, help to mitigate the inflammation. It is necessary to understand the cytokine signatures in COPD due to tobacco and biomass smoke, so a sub-sample of this cohort was evaluated to investigate differences in the cytokine signatures of tobacco smokerelated COPD and biomass smoke-related COPD.

## STUDY PARTICIPANTS

**Figure 1.**
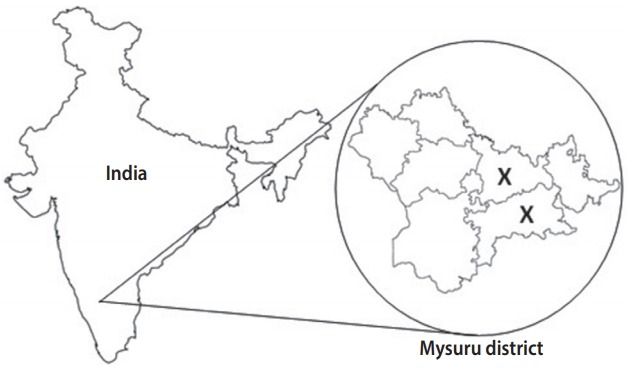
Map of India illustrating the site of sampling from which the Mysuru stUdies of Determinants of Health in Rural Adults (MUDHRA) cohort was set up (X depicts the 2 rural sub-districts selected for the study).

Of the 7 taluks (sub-districts) in the district of Mysuru, 2 were randomly selected: rural Mysuru with 176 villages and Nanjangud with 131 villages. According to the 2001 Registrar General India census [7], each of these villages had 1,800-2,200 men and women above 30 years of age who were potentially eligible for recruitment. The sample size estimation indicated that we needed to screen at least 8,000 men and women aged above 30 years from these villages to obtain approximately 1,000 cases of symptomatic chronic chest disease. This estimation was based on an estimated COPD prevalence of 5% in this population, with 80% power and 10% standard error. Therefore, of the 307 villages, 8 villages from Mysuru and 8 villages from Nanjangud were randomly selected. Figure 1 shows a map of India illustrating the sampling sites from which the MUDHRA cohort was drawn.

Trained field workers conducted a door-to-door survey of all households (n = 3,139) from these 16 villages to identify those above 30 years of age. Houses were visited on at least 3 separate occasions before being declared non-responders (n= 196). Potentially eligible members from each household underwent a standardised structured interview adopted from the Burden of Obstructive Lung Diseases study [30]. This questionnaire was used to obtain information about socio-demographic variables, respiratory symptoms, self-reported diagnosis of respiratory disease and other non-communicable diseases, medical history of consultations with a doctor or hospitalisation, and exposure to risk factors for chest disorders such as biomass fuel smoke and tobacco smoke.

**Figure 2.**
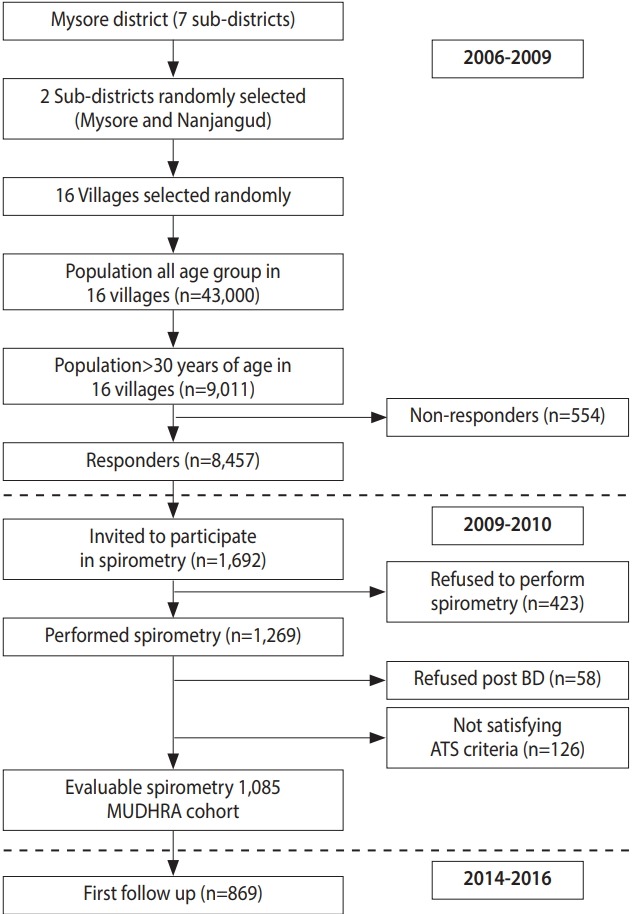
Flowchart for subject sampling and participation in different phases of the study. MUDHRA, Mysuru stUdies of Determinants of Health of Rural Adults; BD, broncho-dilator; ATS, American Thoracic Society.

In the first phase, a total of 8,457 subjects (women: n=3,953, 46.7%; men: n= 4,054, 53.3%) from 3,943 households were screened for symptoms of chronic respiratory diseases and the prevalence of chronic bronchitis. Data were also collected on socioeconomic status, education, occupation, tobacco and alcohol consumption, and biomass smoke exposure. Twenty percent (n= 1,692) of these subjects were randomly invited for further evaluation of lung function and COPD by spirometry. Among 1,692 subjects, 423 declined to participate (62 men and 361 women). More women and men refused to participate due to sociocultural issues. Of the 1,269 subjects who underwent spirometry, 1,085 satisfied the American Thoracic Society (ATS) standards [31]. Except for age and gender, other demographic characteristics were similar between the subjects who did and did not undergo spirometry. These 1,085 men and women constituted the MUDHRA cohort. Figure 2 depicts the flowchart for subject sampling and participation in different phases of the study.

After the baseline assessment for chronic lung disorders and their risk factors during 2006-2010, the cohort was retraced and examined 5 years later between 2014 and 2016. The follow-up evaluation of the cohort was conducted to establish the incidence rates of COPD, its predictors, and other chronic lung disorders. The baseline assessments were repeated and 869 subjects had acceptable spirometry results in the follow-up examination.

During this follow-up, a nested case-control design was used, and a subset of participants (n= 200; 100 with COPD and 100 without) were evaluated further to examine the hypothesis that lower levels of vitamin D and oxidative stress markers and higher levels of depressive symptoms were associated with a greater decline in lung function (manuscript under preparation).

In a smaller subset of tobacco smokers with and without COPD (n = 50), we examined the serum levels of 8 cytokines. Later, in another subset of the cohort (n= 98), we examined the levels and interactions of 40 serum cytokines and chemokines using a multiplex immunoassay system in subjects with COPD related to tobacco smoking (men) and exposure to smoke from biomass fuels (women) compared to subjects exposed to similar levels of risk factors (tobacco smoking in men and biomass smoke exposure in women) but who had not developed COPD to assess whether the immune inflammatory signatures associated with tobacco smoking-related and biomass fuel exposure-related COPD were different (manuscript submitted) [39].

## MEASUREMENTS

The demographic, socioeconomic, health-related, respiratory symptom-related, risk factors, spirometry, and lab variables measured during the different phases are listed in Table 1. Chronic bronchitis was defined as having cough with phlegm on most days for 3 months for at least 2 consecutive years [2]. COPD was defined according to the Global Initiative for Chronic Obstructive Lung Disease (GOLD) spirometry guidelines as a post-bronchodilator ratio of forced expiratory volume to forced lung capacity (FEV1/FVC) < 0.7 [2], and asthma was defined according to the Global Initiative for Asthma spirometry guidelines as an increase of 12.0% and 200 mL in FEV1 on a post-bronchodilator spirometry test [40].

## KEY FINDINGS

Of the 8,457 men and women screened for establishing this cohort, the prevalence of cough and phlegm for a 1-month duration was 14.3%, that of chronic cough and phlegm (for 3 months’ duration) was 8.4%, and that of chronic bronchitis was 7.7%. These conditions were all more common among the elderly, men, and current smokers [41-43].

A threshold of biomass fuel exposure for an elevated risk of the diagnosis of chronic bronchitis was established for the first time in a rural population. The biomass exposure index was first described by Behera & Jindal [44], and was defined as the average hours of exposure to biomass smoke in a day multiplied by the number of years of exposure. We identified that a minimum biomass exposure index of 60 was necessary to have a significantly higher risk for developing chronic bronchitis than the general population. Increased exposure to biomass smoke increased the rates of chronic bronchitis in women [42]. Additional respiratory risk factors, such as an occupation involving contact with dust, were observed in 58.2% of subjects, and passive smoking was reported by 10.9%.

Studies have shown that biomass smoke exposure is a significant factor rivalling tobacco smoke both for the development of COPD and for mortality associated with COPD [45,46]. Increased biomass smoke exposure was associated with increasing severity of airflow limitation and advanced COPD according to the GOLD criteria [47]. The importance of biomass smoke in COPD has been further confirmed by studies that have longitudinally evaluated the effect of switching over to cleaner fuel as compared to continuing biomass smoke exposure and observed more than a 50% decrease in the risk of developing COPD [48].

Indoor levels of carbon monoxide (CO), sulphur dioxide (SO_2_), and nitric oxide (NO) during cooking and 3 hours thereafter were measured in 50 randomly chosen participant households. The levels of all these compounds were unacceptably high (peak CO: 999 parts per million [ppm], with a time-weighted average [TWA] of 596 ppm; peak SO_2_: 99.9 ppm, with a TWA of 23.4 ppm; peak NO level: 38.3 ppm, with a TWA of 5.7 ppm). At 3 hours, the levels of CO, SO_2_, and NO remained unacceptably high at 37, 15, and 4 ppm, respectively (manuscript under preparation).

Of the 1,085 constituent members of the cohort, 9 of the 915 men (1.0%) and 1 of the 170 women (0.6%) were diagnosed with COPD. The most common abnormality on spirometry in both gender was a restrictive defect (40.0%), and this was significantly more common among women than among men in those aged less than 40 years (28.0% of men vs. 43.0% in women). The prevalence of asthma confirmed by spirometry was 6.9% (75 of 1,085) in our study (manuscript under preparation).

At the 5-year follow-up, 72 of the 573 participants (12.6%) with chronic bronchitis and 17 of the 296 participants (5.7%) without chronic bronchitis had died, indicating a much higher all-cause 5-year mortality rate in those with chronic bronchitis (manuscript under preparation).

The nested case-control study (n=98) identified the chemokines chemokine (C-C motif) ligand (CCL)20, CCL27, and chemokine (CX-C motif) ligand (CXCL)13 as putative, plausibly homeostatic biomarkers for biomass smoke-induced COPD. Ten cytokines and chemokines exhibited higher concentrations in the tobacco smokeexposed controls than in the tobacco smoke-exposed COPD cases. A comparison of cytokine and chemokine concentrations between the biomass smoke-induced COPD subjects and the tobacco smoke-induced COPD subjects and the corresponding subjects who were exposed to biomass smoke or tobacco smoke but did not develop COPD also revealed distinct molecular profiles [49] (manuscript submitted).

## STRENGTHS AND WEAKNESSES

The main strength of the MUDHRA cohort is that it is population-based and representative of the rural population in southern India. This is the single largest rural cohort in India in which all participants underwent standardized assessments for the diagnosis of COPD according to ATS criteria, with a repeat assessment for lung function at a 5-year follow-up. Very few declined to participate at baseline (< 5%) and in the follow-up studies (< 7%). We measured CO, SO_2_ and NO in a subset of households. Particulate matter levels (PM_2.5_, PM_10_) were not measured. Participants were not examined for cardio-metabolic disorders. Although all-cause mortality was reported for the cohort at the 5-year follow-up, the cause of death was not ascertained. We intend to overcome this by establishing the probable cause of death by conducting a standardised verbal autopsy interview with a reliable informant of the deceased and recording the cause of death from medical records or the death certificate.

## DATA ACCESSIBILITY

The study data are not freely available, but the MUDHRA cohort team would welcome collaborations with other researchers. For further information, contact Dr. Mahesh PA based at JSS Medical College, JSS Academy of Higher Education and Research, Mysuru, India (mahesh1971in@yahoo.com).

## Figures and Tables

**Table 1.** The list of variables collected during the different phases of the study

Dataset		Available data
Baseline		
Total(n)	8,457	Demographics: age, sex, village, taluk
Age (yr)	30-105	Socioeconomic indicators: education, occupation, type of house, number of people at home, number of room at home, land ownership, vehicle ownership, type of latrine, source of drinking water, presence of indoor animals
Mean±SD (yr)	45.5±12.5	
Year	2006-2009	Health related (respiratory symptoms) cough, phlegm, breathlessness, wheeze
		Self-reported diagnosis of respiratory diseases: emphysema, asthma, allergic bronchitis, chronic bronchitis, COPD, lung cancer, tuberculosis
		Self-reported diagnosis of non-communicable diseases: hypertension, diabetes mellitus, stroke, heart disease
		Medical history of consultation with doctor or hospitalization: consulted or admitted for breathlessness, days of hospitalization, chest surgeries, hospitalization in childhood for respiratory illness
		Risk factors for chest disorders: tobacco smoking: type of smoking (beedi, cigarette, both), age of start, years of smoking, frequency, current smoking status, pack years, smoking index
		Biomass fuel smoke exposure: years of exposure, hours exposed in a day, biomass index
		Environmental exposure to dust at work, tobacco smoke
		Type of fuel used for domestic work (kerosene, gas, biomass) presence of chimney and early life events
		Knowledge and attitudes of smokers towards tobacco smoking and nicotine dependence: Fagerstrom questionnaire (subset n=900) [8,32]
MUDHRA cohort		
Total (n)	1,085	Height, weight, body mass index
Age (yr)	35-80	Lung function: spirometry pre- and post-bronchodilator forced vital capacity, forced expiratory volume in the first second
Mean±SD (yr)	49.9±10.5	
Year	2009-2010	
First follow up		
Total (n)	869	Respiratory symptom severity indicators: COPD Assessment Test [33], St. Georg’s Respiratory Questionnaire [34], modified Medical Research Council dyspnoea scale [35]
Age (yr)	43-90	
Mean±SD (yr)	60.7±12.9	Assessment of physical activity/exercise capacity: six-minute walk distance [36]
Year	2014-2016	Lung function test (spirometry) pre- and post-bronchodilator forced vital capacity, forced expiratory volume in the first second
		Mental health: HAM-A [37] and HAM-D [38] scales
		Serum vitamin D levels (n=200)
		Serum antioxidant reserves thiobarbituric acid reactive substances and ferric reducing antioxidant power (n=200)
		Forty serum cytokines and chemokines (n=98)

SD, standard deviation; MUDHRA, Mysuru stUdies of Determinants of Health in Rural Adults; COPD, chronic obstructive pulmonary disease; HAM, Hamilton Anxiety and Depression.
